# Characterization of Zebrafish Green Cone Photoresponse Recorded with Pressure-Polished Patch Pipettes, Yielding Efficient Intracellular Dialysis

**DOI:** 10.1371/journal.pone.0141727

**Published:** 2015-10-29

**Authors:** Marco Aquila, Mascia Benedusi, Anna Fasoli, Giorgio Rispoli

**Affiliations:** Department of Life Science and Biotechnology, University of Ferrara, Ferrara, Italy; Dalhousie University, CANADA

## Abstract

The phototransduction enzymatic cascade in cones is less understood than in rods, and the zebrafish is an ideal model with which to investigate vertebrate and human vision. Therefore, here, for the first time, the zebrafish green cone photoresponse is characterized also to obtain a firm basis for evaluating how it is modulated by exogenous molecules. To this aim, a powerful method was developed to obtain long-lasting recordings with low access resistance, employing pressure-polished patch pipettes. This method also enabled fast, efficient delivery of molecules via a perfusion system coupled with pulled quartz or plastic perfusion tubes, inserted very close to the enlarged pipette tip. Sub-saturating flashes elicited responses in different cells with similar rising phase kinetics but with very different recovery kinetics, suggesting the existence of physiologically distinct cones having different Ca^2+^ dynamics. Theoretical considerations demonstrate that the different recovery kinetics can be modelled by simulating changes in the Ca^2+^-buffering capacity of the outer segment. Importantly, the Ca^2+^-buffer action preserves the fast response rising phase, when the Ca^2+^-dependent negative feedback is activated by the light-induced decline in intracellular Ca^2+^.

## Introduction

G-protein-coupled enzyme cascades are ubiquitous signalling systems: they transduce physical or chemical stimuli, triggered by photon absorption or ligand binding (e.g. ions, hormones, neurotransmitters, or odorants), into intracellular messages that elicit adaptive responses [1, 2]. The most direct, most sensitive method for studying these cascades is to precisely control the stimulus with light (for instance, by using optogenetics or caged agonists [3–5]), and recording the stimulus response with electrophysiological methods which are suitable for those systems that target ion channels. The study of how changes in specific transduction proteins − naturally occurring or produced by site-directed artificial mutants (as well as by knock down or over-expressing mutants) − affect electrical response is a potent method with which to investigate their functional role in vivo or ex vivo [6–9]. However, the production of a vital transgenic animal is often expensive and time consuming; furthermore, the protein level alteration is permanent, i.e. irreversible, and often, due to the activation of compensatory pathways, the genetic manipulation does not isolate the specific function of the protein under study [10].

Vertebrate visual phototransduction (reviewed in [11–14]) is one of the most extensively studied G-protein cascades and represents a powerful model system for investigating G-protein signalling, the advantage being that it is “naturally” activated by light. It is also important to understand animal vision because it is ultimately limited by photoreceptor performance. Vertebrate vision is initiated by the light-induced, transient suppression of the current flowing into the outer segment of the photoreceptor through the cGMP-gated channels in its plasma membrane. Changes in the intracellular cGMP concentration can be monitored in real time by whole-cell or suction electrode recording, the most accurate probe of functional integrity and performance of the phototransduction cascade and of the effect specific intracellular manipulations can exert on its enzymes [15–17]. Moreover, the outer segment contains high concentrations of proteins involved in this cascade (compared with proteins of other enzymatic processes) and thus they can be purified for biochemical, protein interaction and functionality studies [12, 18]. Combined with electrophysiological experiments, these assays have provided a precise, quantitative analysis of visual enzymatic processes, also permitting the realization of functional mathematical models [19–21].

Here we present a new method for the ex vivo study of a G-protein coupled enzymatic cascade, the ultimate goal being to up- and down-regulate a photoreceptor protein and, in parallel, measure how the light response is affected. An obvious strategy for realizing this is, for instance, to deliver the specific protein itself, or its monoclonal antibody, during whole-cell recording of photoresponses, here performed for the first time on green-sensitive cones of zebrafish. This animal has attained increasing interest in recent years as a model for the investigation of vertebrate and even human vision [22]. Moreover, its embryonic development is remarkably fast [23] and huge resources for its genetic engineering are available. We narrowed our research to green cones because they can be isolated from the zebrafish retina in larger numbers than the other cone types. Finally, since phototransduction in cones is not as well understood as in rods [24], we also investigated some basic properties of cone cell responses. Whole-cell recording with intracellular perfusion was then developed for these very small cells because it circumvents many problems affecting other recording techniques. For instance, suction-pipette recordings from zebrafish cones [25, 26] do not allow the investigator to collect the entire membrane current, to clamp the membrane voltage, or to manipulate the intracellular milieu. To some extent, suction recordings from truncated photoreceptors [27] circumvent the last problem only, but they do carry a high risk of losing cytoplasmic molecules. Truncation of outer segments smaller than those from amphibians is even more demanding because it would lead to severe mechanical cell injury and poor signal-to-noise ratios. However, whole-cell recording from such minute cells as zebrafish cones requires patch pipettes with a very small tip opening: their conventional fabrication inevitably produces very long and tapered shanks, leading to high access resistance, low diffusional rate between pipette and cytosol, and makes inserting perfusion tubes in the pipette lumen infeasible.

Here, the exogenous molecules were administered during whole-cell recording by using pressure-polished patch pipettes [28] whose enlarged shank could accommodate pulled perfusion tubes very close to the pipette tip, and greatly reduced the access resistance. To provide the fast, controlled cytosolic incorporation of exogenous molecules, the intrapipette tube was coupled to a perfusion system. In the following, the efficacy of the intrapipette perfusion apparatus was tested by measuring the rate of accumulation of a fluorescent dye, injected in the outer segment or in the inner segment cytosol during whole-cell experiments. The whole-cell recordings of the photoresponse of green-sensitive cones, employing pressure-polished pipettes, were then analyzed in details for the first time, thus establishing the performance of these cells under control conditions. Analysis of the photoresponse recovery phase suggests the existence of physiologically distinct cones having different Ca^2+^ dynamics. The Ca^2+^-buffering capacity of the outer segment strongly regulates this dynamics: importantly, a minimal model fitting the photoresponses demonstrates that this Ca^2+^-buffer prevents the slow-down that can occur in the response rising phase when a negative feedback (whatever its nature) regulating receptor sensitivity is activated during the light response. Finally, the extremely fast rising phase of the response to supersaturating flashes is consistent with the hypothesis that rhodopsin may interact with transducin in the dark by forming preassembled complexes [29].

## Materials and Methods

### Cone Isolation and Viewing

All experiments on zebrafish (*Danio rerio*) were performed in compliance with the European Communities Council Directive for animal use in science (86/609/EEC) and approved by the ethical committee of the University of Ferrara named “Comitato Etico di Ateneo per la Sperimentazione Animale” (C.E.A.S.A.)”. Animals 4–5 cm in length were purchased from a local supplier, with no more than 30 specimens maintained in a 70-liter tank containing recirculated water continuously filtered and aerated, at 26–28°C on a 12 h light—12 h dark cycle, following the guidelines reported in [30].

A healthy zebrafish was dark-adapted for 3 h and sacrificed in dim red light (generated by LEDs with wavelength of 660 nm) by cranial concussion followed by decapitation and pithing. The head was swiftly immersed in a Petri dish containing Ringer solution of the following composition (in mM): 115 NaCl, 3 KCl, 10 HEPES free acid [*N*-(2-hydroxyethyl)piperazine-*N’*-(2-ethanesulfonic acid)], 0.6 MgCl_2_, 0.6 MgSO_4_, 1.5 CaCl_2_, 10 glucose (buffered to pH = 7.6 with NaOH; osmolality: 260 mM mOsm/Kg). The Petri dish containing the head was transferred in a fully darkened box equipped with infrared LEDs (wavelength: 940 nm) and a high definition webcam that had its infrared filter removed and was connected to an external monitor. After inserting the hands through the two light-tight holes of the box, the cornea of each eye was focussed on the monitor screen and it was cut with a razor blade piece. Each retina was separated from the pigment epithelium by sucking it gently into the tip of a 200 μl pipette, which was also used to mechanically dissociate the photoreceptors by triturating the retina. An aliquot (~2 ml) of the Ringer solution containing the photoreceptors was transferred to the recording chamber placed on the microscope (TE 300, Nikon, Tokyo, Japan) stage. The preparation was illuminated with a cluster of ultra-bright infrared LED (900 nm) and focused on a fast digital camera (C6790-81, Hamamatsu Photonics, Tokyo, Japan) coupled to the microscope.

### Recordings from Zebrafish Cones and Intracellular Perfusion

Light responses of zebrafish cones were recorded using the whole-cell configuration of the patch-clamp technique, under dark-adapted condition at room temperature (20–22°C). Pipettes were usually sealed on the cone outer segment, unless specified otherwise; details of the patch-clamp apparatus is described elsewhere [28, 31]. The current amplitude elicited by repetitive -10 mV pulses was used to measure the cell capacitance, and the seal, the access (*R*_*a*_), and the membrane resistance; the holding potential was always -40 mV. Whole-cell recordings were low-pass filtered at 2 Khz by an eight-pole Butterworth filter (VBF/8 Kemo, Beckenham, UK), sampled at 19.2 kHz/16 bits by a A/D board (Digidata 1322A; Molecular Devices, Sunnyvale, CA, USA) controlled by Clampex software (Molecular Devices), and analyzed with Clampfit (Molecular Devices), Sigmaplot (Systat Software Inc., San Jose, CA, USA) and Mathcad (Parametric Technology Corporation, Needham, MA, USA) software. To reject as much noise as possible, each photoresponse shown in the figures was the average of three or more responses to the same flash; this average response was then further filtered by using a local smoothing routine implemented in the commercially available program Sigmaplot. The routine parameters were set to obtain low-pass filtering of 30 Hz (negative exponential was selected for smoother function, with a sampling proportion of 0.01, a polynomial degree 1, and a number of interval that was 1/10 of the total number of samples constituting each trace, i.e. 19220 samples for a 1 s trace). Traces longer than 2 s were just low-pass filtered at 20 Hz with the Gaussian filter of the pClamp routine, since they illustrate only slow aspects of the photoresponses: their huge number of points made it impractical to use the Sigmaplot smoothing routine on desktop computers.

Results are given as means±SEM.

**Fig 1:**
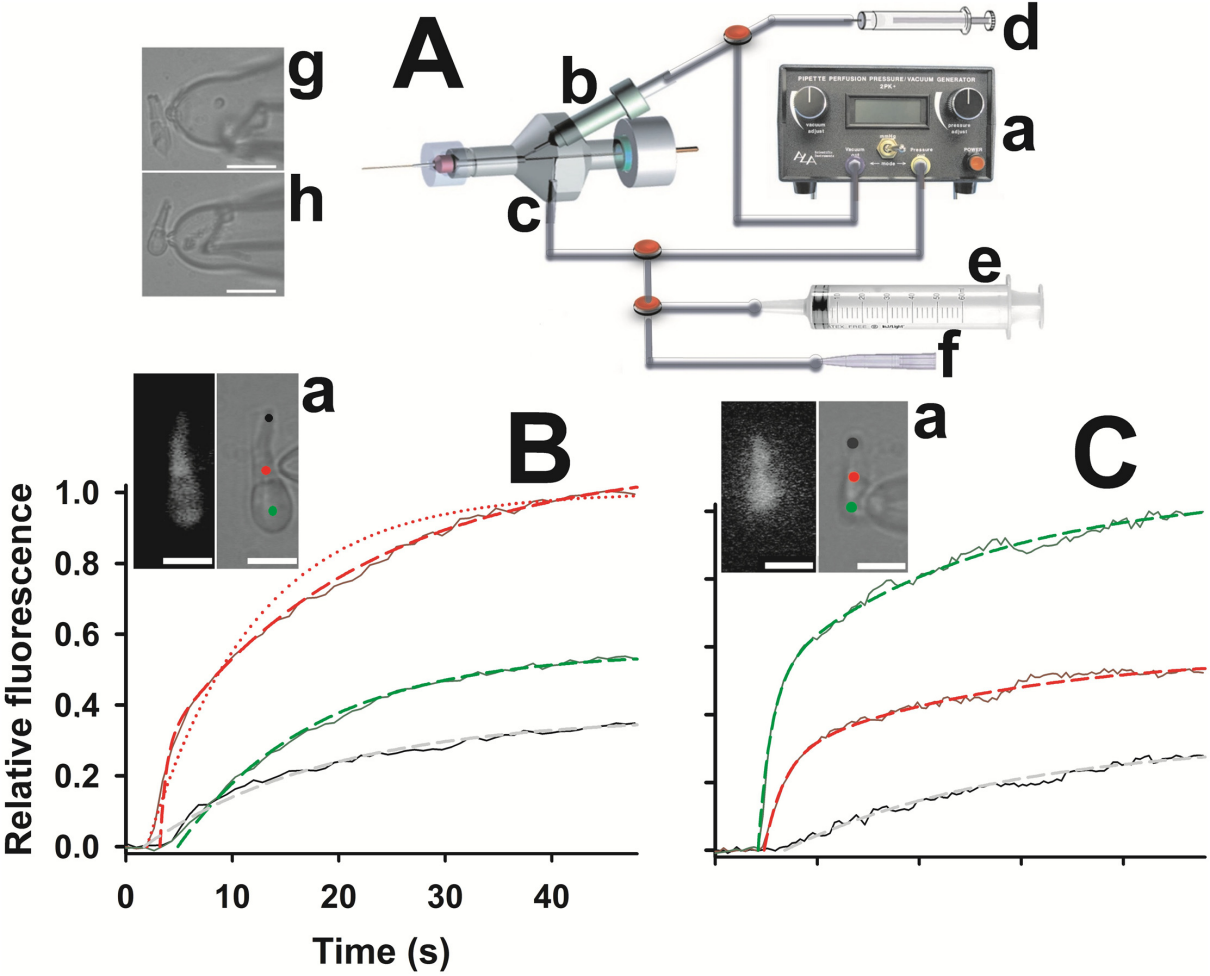
Methods. *A*, The cytosolic perfusion was realized with a commercial instrument (*a*) that applied a positive pressure to the perfusion tube and a simultaneous depression in the standard side-port (*c*); perfusion tube was inserted in the pipette lumen (*g* and *h*; white scale bar is 20 μm) via a custom side-port drilled in the holder, (*b*), and filled with the intracellular solution containing the exogenous molecules. Alternatively, the positive pressure was applied with a precision syringe (*d*) connected to the perfusion tube by means of a three-way valve (*red* disk), whose piston was moved with a micromanipulator (not shown); in this case no depression was applied. The standard side port could also be connected, with two three-way valves, to a 50 ml syringe (*e*) to apply strong positive pressure to clean the pipette, or to a mouth piece (*f*), to attain the seal on the cone outer segment (*g* and *B*) or on its inner segment (*h* and *C*). *B*, fluorescence intensity vs time of lucifer yellow (40 μM, injected at time 0 in the outer segment, *a*; *white* scale bars are 10 μm; *R*_*a*_~4.1 MΩ), integrated in the *black*, *red*, and *green* regions of *a*, *right* panel, normalized to the maximal intensity recorded in the site of dye injection (*red* region). Data (*black*, *dark red* and *dark green* curves in *b*) are fitted with a biexponential equation (*red dashed* line, *τ*_*f*_ = 0.6 s, *A*_*f*_ = 0.29, *τ*_*s*_ = 19 s, *A*_*s*_ = 0.8) and with a monoexponential one (*grey dashed* line, *τ*_*s*_ = 17.5 s, *A*_*s*_ = 0.37; *red dotted* line, *τ*_*s*_ = 10 s, *A*_*s*_ = 1; *green dashed* line, *τ*_*s*_ = 13 s, *A*_*s*_ = 0.55). Fluorescence image after 50 s of lucifer yellow perfusion is shown in *a*, *left* panel. *C*, same experiment of *A* on a morphologically different green cone, with the patch pipette sealed on the inner segment (*a*; *R*_*a*_~6.6 MΩ); data (same colour coding of *A*) are fitted with a biexponential equation (*red dashed* line, *τ*_*f*_ = 1.9 s, *A*_*f*_ = 0.27, *τ*_*s*_ = 22 s, *A*_*s*_ = 0.31; *green dashed* line, *τ*_*f*_ = 1.3 s, *A*_*f*_ = 0.5, *τ*_*s*_ = 20 s, *A*_*s*_ = 0.56) and with a monoexponential one (*grey dashed* line, *τ*_*s*_ = 23 s, *A*_*s*_ = 0.33).

Patch pipettes were filled with an intracellular solution containing (in mM): 40 KCl, 70 K-Asp, 5 MgCl_2_, 1 GTP, 5 ATP, 5 HEPES (buffered to pH = 7.6 with KOH; osmolality: 260 mM mOsm/Kg). All chemicals were purchased from Sigma (St. Louis, MO, USA). The intracellular dialysis of zebrafish cones was performed by using pressure-polished pipette and an intrapipette perfusion system (Fig 1A). Besides improving the electrical recordings, the enlarged shank of pressure-polished pipettes [28, 31] can accommodate pulled quartz or plastic perfusion tubes close to the pipette tip (Fig 1A), allowing the fast and controlled cytosolic incorporation of exogenous molecules. The perfusion tube was filled with an intracellular solution containing the molecules under study, which was injected in the cytosol during whole-cell recording by the controlled application of pressure to the capillary lumen. The pressure was delivered by a commercially available perfusion pressure/vacuum generator (2PK+, ALA scientific instruments, New York, New York; applied pressure: ~40 PSI (~280kPa)), or with a 1 ml precision syringe coupled to a micromanipulator (Fig 1A).

### Light Stimulation and Calibration

Light stimuli (flashes in the dark and flashes on a background of light) were designed using the voltage protocols of Clampex software. One of the two analogical outputs of the Digidata board was connected to a calibrated voltage-to-current converter driving an ultra-bright trichromatic LED (with center wavelength red/green/blue of 627.5±8/525±10/467.5±8 nm), that had its plastic lens replaced with a frosted filter. The LED was mounted on a miniaturized *xyz* micromanipulator and coupled to the binocular port of the inverted microscope (Nikon TE-300, Tokyo, Japan). The LED was aligned so as to have its light spot automatically centered and in focus on the zebrafish cone, when the latter, illuminated with the cluster of ultra-bright infrared LEDs, was viewed in sharp focus through a 60x objective on the microscope camera (C6790-81, Hamamatsu Photonics, Tokyo, Japan). Enough space was left between the trichromatic LED and the binocular port to host neutral density filters, when required; the trichromatic LED positioning and measurement of its spot size were performed with the following procedure. First, a particular subdivision of the grid of a calibrated microscope slide was focused and centered on the inverted microscope 60x objective, by using the microscope infrared LED illumination. Then a straight microscope, mounted on a second *xyz* micromanipulator, was aligned to the inverted one, and it was moved so its 60x objective was focused and centered on the same grid subdivision using the trichromatic LED as a light source. The LED spot, observed with the straight microscope, was finally focused and centered with its *xyz* manipulator on the grid subdivision, and its geometry measured (it resulted a uniform disk of 300 μm of diameter). The microscope slide was then replaced by the sensor (OP-2/LM-2 VIS, Coherent, Santa Clara, CA, USA) of a power/energy meter (Fieldmaster, Coherent), and the light power produced by the green and the red section of the trichromatic LED was measured at all the voltages used in the experiments. Voltages, neutral density filters, and light stimulus duration were matched so as i) to have a wide range of light intensities spanning more than 5 order of magnitude, with currents always falling in the green and red LED linear range (1–50 mA) and ii) to have the same light intensity of red and green LED (the blue one was not used). The duration of all flashes up to 1.77·10^4^ photons/μm^2^ of intensity was 1 ms, above this intensity it was 10 ms.

To estimate the physical collecting area of the zebrafish cone outer segment (the functional collecting area cannot be estimated since cones do not detect single photons), the quantum efficiency of photoisomerization and the specific axial rhodopsin density must be known [32]. Moreover, it is difficult to have a reasonable estimate of the cone rod outer segment volume, since it is strongly invaginated, its geometry cannot be simply assimilated to a truncated cone, and its small dimension makes precisely measuring the diameter of base and tip difficult. The geometrical area of the cone outer segment was measured on digital micrographs obtained with the camera (494x674 pixels, 0.2 μm per pixel) using a freeware image analysis software (ImageJ, http://rsb.info.nih.gov/ij/) and was 15.3±3.9 μm^2^ (*n* = 20). Using the ratio between the geometrical rod outer segment area and the collecting area of ~0.062 calculated by Baylor et al., 1979, it can be roughly estimated that the zebrafish cone collecting area is ~1 μm^2^. If this line of reasoning proves to be correct, then the numerical values of the flash intensities reported throughout the paper (in photons/μm^2^) are also the number of photons absorbed by the cone.

### Fluorescence Imaging

Besides bright-field viewing of the cells, the camera and its controlling software (AquaCosmos, version 2.5.3.0; Hamamatsu Photonics) were also employed for fluorescence imaging experiments; the excitation light was generated by a monochromator (Polychrome II, Till Photonics, FEI, Hillsboro, Oregon, USA) coupled to the epifluorescence port of the microscope via an optical fiber. Solution loading in the cell was checked by using lucifer yellow (CH, dilithium salt; dissolved in intracellular solution at 40 μM concentration; excitation: 425 nm, emission: 528 nm; Fig 1B and 1C). Image analysis was performed by using AquaCosmos and MATLAB (MathWorks, Natick, MA, USA) software. The effectiveness of the molecular cytosolic loading during whole-cell recordings was assessed by measuring the dynamics of fluorescence rise integrated in three cellular regions (one close to the patch pipette and two far away from it, Fig 1B, panel *a* and Fig 1C, panel *a*), following the injection of lucifer yellow into the cell. In principle, many factors influence such dynamics, for example cell geometry, where the pipette is sealed on the cell, the effective pressure applied inside the pipette perfusion tube, the position of this tube inside the pipette, pipette geometry and access resistance (*R*_*a*_). Despite all these factors, fluorescence grew with similar kinetics in all cells examined: in the regions close to the pipette, fluorescence dynamics were described by the sum of a fast (parameters with the subscript *f*) exponential component and a slower (subscript *s*) one:

$$F\left(t\right)=A_{f}\left(1-e^{-\;\frac{t}{\tau_{f}}}\right)+A_{s}\left(1-e^{-\;\frac{t}{\tau_{s}}}\right)$$

The fast component amplitude (i.e. *A*_*f*_) progressively decreased (i.e. it was “low-pass filtered”) as the distance between the pipette (i.e. the point of dye injection) and the integration region was larger. Eventually, the fluorescence increased in the distal regions (with a small delay that was never larger than 5 s) with a slow monoexponential kinetics (Fig 1B and 1C). Therefore, the parameters that were reproducible from cell to cell were *τ*_*f*_ in proximity of the pipette and *τ*_*s*_ in all points (*τ*_*f*_~0.97±0.18, *τ*_*s*_~18.3±0.7; *n* = 11). As an example, Fig 1 reports the fluorescence dynamics and the relative fittings in the two most different recordings obtained: a large cell, where the patch pipette was sealed on the outer segment (Fig 1B), and a much smaller cell, with a very different morphology, where the patch pipette was sealed on the inner segment (Fig 1C). It can be concluded that, in all cone regions, a substantial cell loading of an exogenous molecule is achieved one minute after molecule injection.

## Results

**Fig 2:**
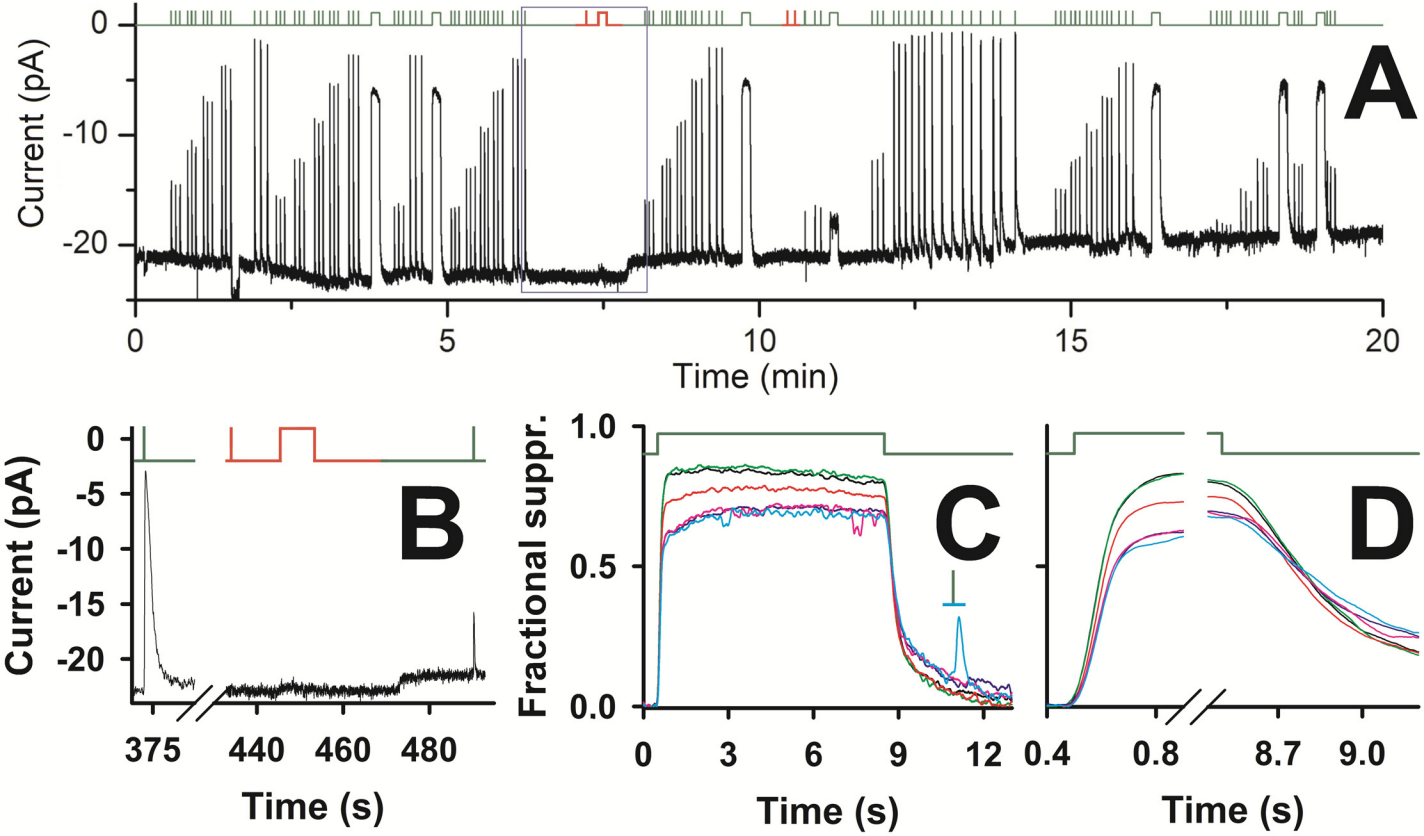
Recording stability and wavelength discrimination of the zebrafish green sensitive cones. *Upper* panel of *A*-*D*, timing of delivery of green (525 nm, *dark green* lines) and red (627.5 nm, *red* lines) flashes and steps of light. *A*, *lower* panel, whole-cell recording (*R*_*a*_~6.0 MΩ) of the current of a green sensitive cone on a very slow time scale; the zero is the level measured once seal has been achieved (holding potential: -40 mV) at the beginning of recording (corresponding to zero time), and not the level corresponding to full suppression of the light sensitive current. *B*, enlargement of the recording in the blue box in *A*, where a sequence of a nearly saturating green flash (1.77·10^4^ photons/μm^2^), a red flash (1.14·10^3^ photons/μm^2^, that was delivered twice again after the green step at 583 s), a red 8 s step (2.5·10^4^ photons/(μm^2^·sec)), and a green flash (1.14·10^3^ photons/μm^2^) were delivered. *C*, normalized responses to 8 s green steps (1.8·10^5^ photons/(μm^2^·sec)) delivered at times: 227 s (black trace), 285 s (green), 583 s (red), 977 s (blue), 1100 s (pink), and 1136 s (cyan); the cyan and green line is the timing of a green flash (1.14·10^3^ photons/μm^2^) delivered during the recovery phase of the step response at 1136 s. *D*, enlargement of the rising and of the falling phase of the step responses of *C*.

To perform whole-cell recordings from very small and fragile cells as the zebrafish cones, a particular patch pipette shape was designed (as described in [28, 31]), characterized by a very small tip and an enlarged shank, which allowed low access resistance (*R*_*a*_~6.7±1.1 MΩ range: 2.0–13.0 MΩ *n* = 36) recordings and efficient cytosolic incorporation of exogenous molecules. Long lasting recordings (Fig 2A) with pressure-polished pipettes (Fig 1) were obtained more often from isolated green cones, therefore this study was limited to this cell type only.

**Fig 3:**
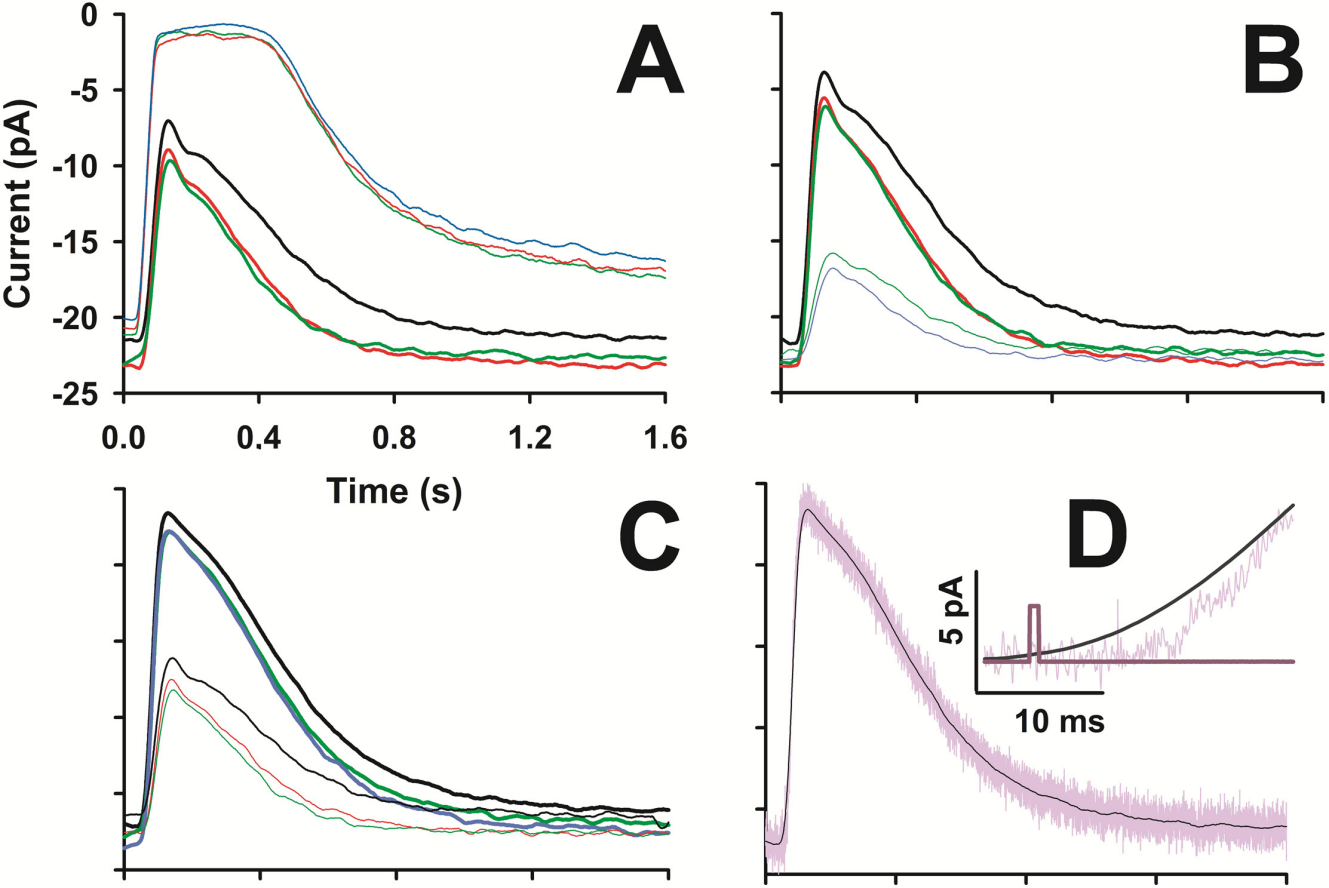
Flash response reproducibility. *A*, *B*, and *C* illustrate the average and smoothed response (as described in Methods) of three consecutive flashes of same intensity delivered within ~2, ~5, ~10, ~15 min from the beginning of recording (*black*, *green*, *red*, and *blue* traces, respectively) of six different intensities (in photons/μm^2^), grouped in two intensities in each panel: 4.49·10^3^ and 3.76·10^5^ (*A*), 1.14·10^3^ and 8.68·10^3^ (*B*), 2.32·10^3^ and 1.77·10^4^ (*C*). All responses were aligned with the flash delivery time, but not along the ordinate (that is the same for all three panels), therefore the zero is the same as Fig 2A. *D*, the unsmoothed average of three consecutive responses to a nearly saturating flash (1.77·10^4^ photons/μm^2^, *purple* trace) is compared to the smoothed one (*black* trace), enlarged in the *inset*; the flash timing is indicated in *dark purple*.

**Fig 4:**
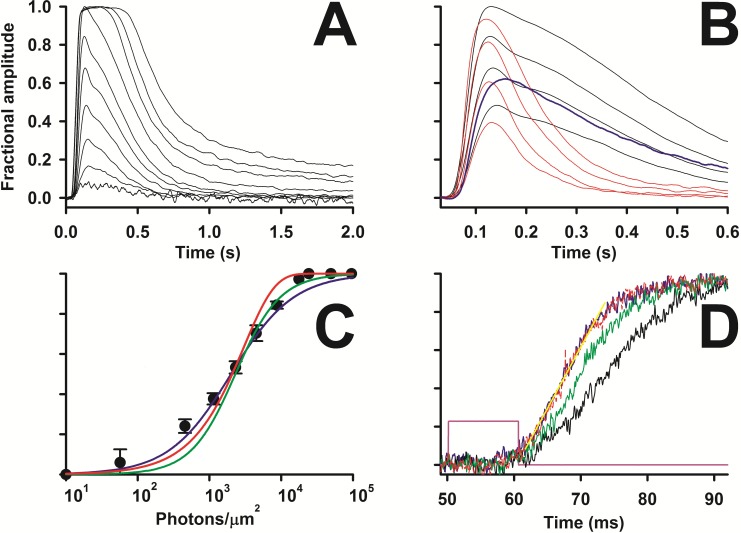
Flash response waveforms. The responses reported in panels *A*, *B*, and *C* of Fig 3 were averaged together and the corresponding six traces were aligned and normalized in panel *A*. To these, four other responses were added to flashes delivering 1.14·10^2^ (9 responses averaged), 1.16·10^3^, 9.55·10^4^, and 1.85·10^5^ photons/μm^2^. *B*, Response waveform had either a prominent dual component recovery (*black* traces, that are the responses to flashes of 2.32·10^3^, 4.49·10^3^, 8.68·10^3^, and 1.77·10^4^ photons/μm^2^ of panel *A*), or had a single kinetic component recovery (*red* traces, responses from a different cell to the same flash intensities), or a recovery in between these two types (*thick blue* trace). *C*, response amplitude vs light intensity (each data point, *black* dots and error bars, is the average of at least 75 responses to the same flash; *n* = 25). Hill equation fit (*m* = 1, *I*_*0*_ = 2·10^3^ photons/μm^2^, *blue* trace; *m* = 1.4, *I*_*0*_ = 2.3·10^3^ photons/μm^2^, *green* trace) and exponential saturation equation fit (*I*_*0*_ = 3·10^3^ photons/μm^2^, *red* trace) to the data points. *D*, *black*, *green*, *red*, and *blue* traces are the responses (of panel *A*) to flashes delivering 1.77·10^4^, 9.55·10^4^, 1.85·10^5^, and 3.76·10^5^ photons/μm^2^, respectively, but unsmoothed; *yellow* trace is the linear fit to the rising phase of the two fastest responses (evoked by flashes delivering 1.85·10^5^ and 3.76·10^5^ photons/μm^2^) having a slope of 64 s^-1^ (correlation coefficient: 0.97); flash timing is the *dark purple* trace.

**Fig 5:**
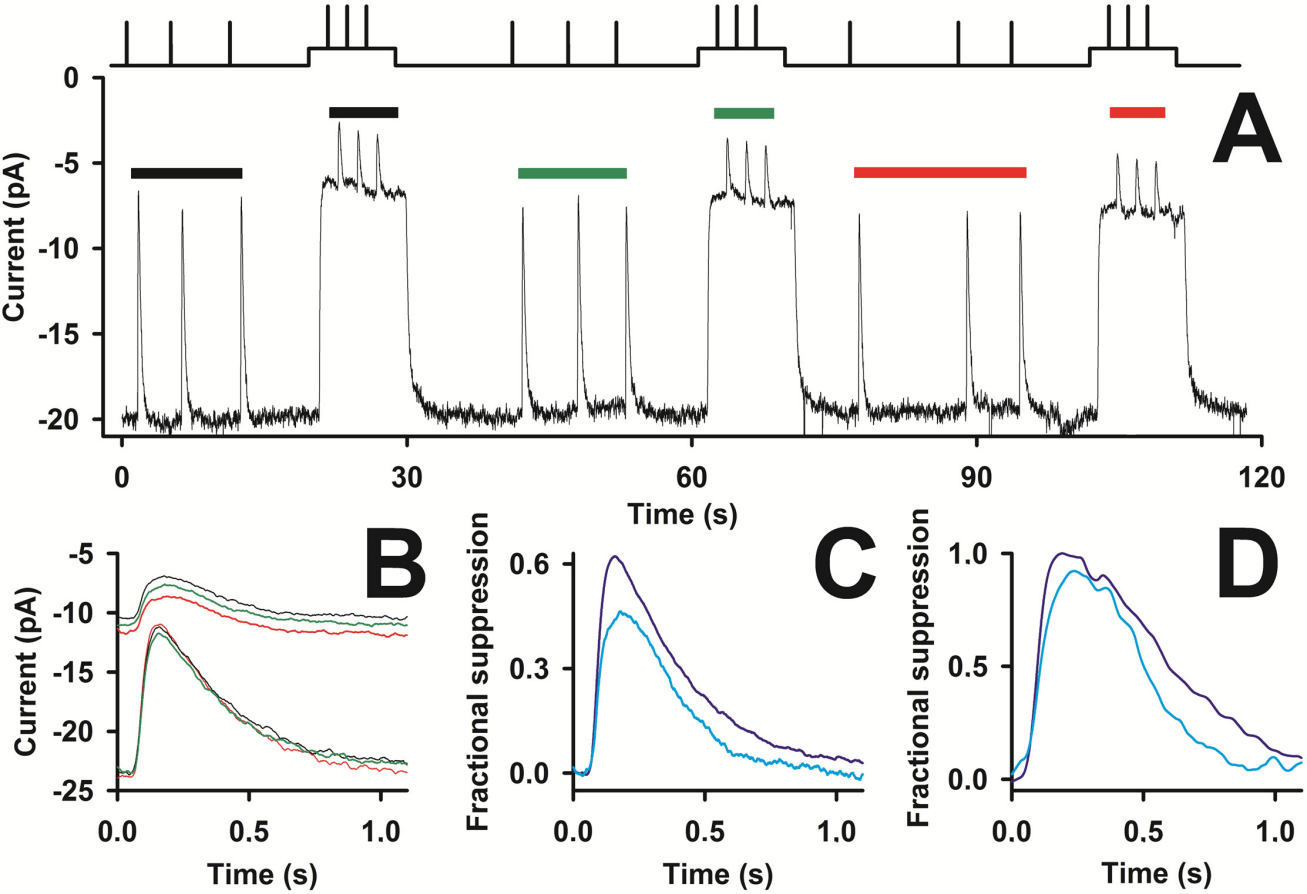
Stability of the light adaptation. *A*, *lower* trace, recording (*R*_*a*_~5.7 MΩ) of the dark current in response to flashes and steps of light whose timing is shown in the *upper* trace; the zero current corresponds to full suppression of the light-sensitive current. *B*, the average of three responses to the same flash (4.49·10^3^ photons/μm^2^), delivered in the dark at the beginning (*black* trace in *B* and *black* bar in *A*), in the middle (*green*), and at the end (*red*) of the recording shown in *A*, is compared to the corresponding average of the three responses to the same flash (8.68·10^3^ photons/μm^2^) delivered on the three identical backgrounds of light (1.3·10^5^ photons/(μm^2^·sec)). *C*, comparison between the average of the 9 flash responses in the dark of *B* (suppressing, on average, ~65% of the dark current, that is ~19.7 pA; *blue* trace), and the average of the 9 flash responses during the three light steps of *B* (suppressing ~45% of the residual dark current of each step, which is ~7.3 pA; cyan trace). *D*, comparison (in another cell) between the response to a 1.77·10^4^ photons/μm^2^ flash (*blue* trace) and the response to the same flash superimposed on the same background of *A* (*cyan* trace).

Since the image resolution of the bright field microscope used could not unambiguously ascertain the morphology of small cells, it seemed likely that some recording might instead be derived from the red cone counterpart of a double red and green cone [33]. To ensure that the recordings presented here were from green cones only, red and green stimuli were routinely delivered (Fig 2A and 2B). As a rationale, a cone is considered green sensitive if its response to a green (525 nm) flash 1.14·10^3^ photons/μm^2^ in intensity suppressed ~40% of the dark current (average suppression: 0.38±0.03, 334 flashes averaged in 25 cells) but it did not respond to a red flash (627.5 nm, Fig 2A and 2B) of same intensity. A typical recording using pressure-polished pipettes, lasting more than 20 min, is illustrated in Fig 2A on a very slow time scale: light flashes of increasing intensity (range: 1.14·10^2^ to 3.76·10^6^ photons/μm^2^), were delivered in triplets in repeated sequences. The small drift in the baseline was due to small changes in the current flowing through the inner segment channels rather than in changes in the light sensitive (or dark) current flowing through the cGMP channels, because the amount of current suppressed by saturating flashes (with intensities ≥1.8·10^4^ photons/μm^2^) was the same (average: 18.7±1.8 pA; *n* = 25) from the beginning to the end of these recordings. Responses to the same flash intensities were averaged and smoothed (see below and Methods): they were reproducible over a recording time of up to more than 20 min. This reproducibility is exemplified by the recording of Fig 3A–3C: consecutive flashes of the same intensities, delivered within ~2, ~5 ~10 and ~15 min from the beginning of whole-cell recording, did not reveal any significant differences with respect to dark current amplitude and response kinetics. Therefore these responses were all averaged, aligned, and normalized as shown in Fig 4A and 4B. Steps of light (1.8·10^5^ photons/(μm^2^·sec), lasting 8 s) were occasionally delivered during this recording (Fig 2A, 2C and 2D) at the following times: 227 s (black trace), 285 s (green), 583 s (red), 977 s (blue), 1100 s (pink), and 1136 s (cyan). These steps gave a constant fractional suppression of 83% (averaging the response between 2.5 and 6 s, where current was most stable) for at least 5 min of recording, and declined to 69% (Fig 2C) after 20 min, but after having delivered repetitive supersaturating flashes (Figs 2A, 4A and 4D). Light sensitive current declined from 20.6 to 19.8 pA during this 20 min recording, i.e. 3.9%, while sensitivity in the same period declined of 17.3%. The decline in sensitivity and dark current was in general not larger than 5% and 10%, respectively, in the first 12.5±1.4 min (*n* = 20; range: 4–23 min) of recording. Responses in Fig 4A recovered from the maximum amplitude with a characteristic waveform, consisting of dual kinetic components, that was particularly evident for flash intensities in the range 2·10^3^ to 10^4^ photons/μm^2^. This feature was observed clearly in ~40% of the recordings (*n* = 25), while others had a faster recovery with a single kinetic component, (Fig 4B, *red* traces, compared with the corresponding four responses with a dual component recovery, in *black*, on an expanded time scale). In fact, a continuum of behavior in between these two recovery types (as the *thick blue* trace, that is the *blue* trace of Fig 5C) was observed.

**Fig 6:**
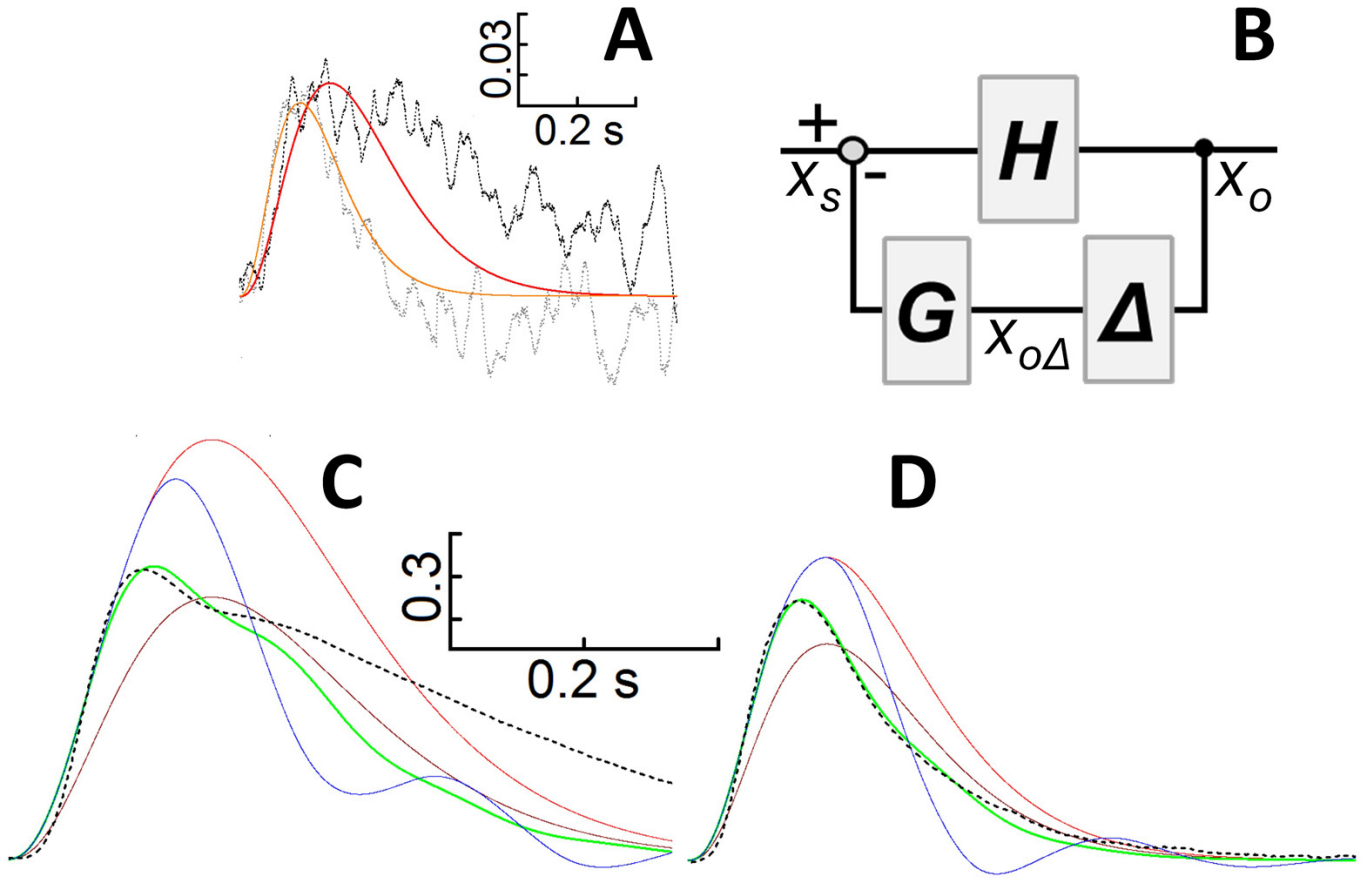
Theoretical prediction of the effect of a Ca^2+^ buffer on the photoresponse recovery phase. *A*, average response (*n* = 9) to the same flash (1.14·10^2^ photons/μm^2^) having a slow kinetics (*black dotted* trace) or a fast one (*grey dotted* trace) fitted by *x*_*s*_ *(t)* with, respectively: *A*_*s*_ = 370, *τ* = 0.052 s; *A*_*s*_ = 1100, *τ* = 0.035 s; these responses are from the two cells of Fig 4B, whose responses are plotted in *black* and in *red*, respectively. *B*, schematic of the “delayed” feedback, the symbols are explained in the text. The gain is set to 1, so *G* is the fraction of the response amplitude “feedbacked” to the input. *C*, *red* trace, plot of *x*_*s*_ *(t)* with *A*_*s*_ = 5200 and *τ* = 0.052 s; *brown* trace, plot of *x*_*o*_ *(t)* (with same *A*_*s*_ and *τ* of *red* trace) with *G* = 0.6 and *Δ* = 0; *green* trace, plot of *x*_*o*_ *(t)* with the same parameters of the *brown* trace but *Δ* = 0.06 s to fit a representative experimental response with a dual kinetic component recovery (to flash of intensity 4.49·10^3^ photons/μm^2^, plotted in *black* in Fig 4B; *dotted black* trace in this panel); parameters of *blue* trace as the *green* trace, but *Δ* = 0.1 s. *D*, responses with the same colour code of *C*: *red* trace, *x*_*s*_ *(t)* with *A*_*s*_ = 12300 and *τ* = 0.035 s; *x*_*o*_ *(t)* is plotted in *brown* with *G* = 0.4 and *Δ* = 0 s, in *green* with *Δ* = 0.055 s, and in *blue* with *Δ* = 0.1 s; the *green* trace fits a representative experimental response with a single kinetic component recovery (to flash of intensity 4.49·10^3^ photons/μm^2^, plotted in *red* in Fig 3B; *dotted black* trace in this panel).

Besides these differences in recovery phase, all green-sensitive cones had similar photoresponse rising time and sensitivity, with a threshold of ~10^2^ photons/μm^2^ (the average response to this flash intensity always had recovery with a single kinetic component; Figs 4A and 6A). Therefore, all response amplitudes were collectively analyzed as a function of the light intensity *I* in Fig 4C. Data were poorly fitted by the Hill equation *R(I)*:

$$R\left(I\right)=\frac{I^{m}}{I^{m}+I_{0}^{m}}$$

where *I*_*0*_ is the half-saturating intensity and *m* is Hill coefficient. This equation fitted the data point at low intensities but not at higher ones (*I*_*0*_ = 2·10^3^ photons/μm^2^, *m* = 1; Fig 4C, *blue* curve), or vice-versa (*I*_*0*_ = 2.3·10^3^ photons/μm^2^, *m* = 1.4; *green* curve). The best fit was given by the exponential saturation equation:

$$R\left(I\right)=1-e^{I/I_{0}}$$

Where *I*_*0*_ = 3·10^3^ photons/μm^2^ (*red* curve).

In order to reduce the background noise, photoresponses were averaged and further processed with a smoothing routine (see Methods). This operation did not alter the entire waveform of the photoresponse to flashes − from dim up to just saturating ones (i.e. delivering less than ~10^4^ photons/μm^2^) − but it slightly altered the rising phase of stronger flashes at early times. Since this resulted in an underestimation of the delay between the flash delivery time and onset of the response (Fig 3D, *inset*), the analysis of the responses to supersaturating flashes were performed on unsmoothed traces (that were then just low-pass filtered at 2 kHz by the anti-aliasing filter; Figs 3D and 4D). The rising phase kinetics accelerated upon increasing the flash intensity up to 1.85·10^5^ photons/μm^2^, but above this value no further acceleration was observed (Fig 4D). The linear fit to the normalized rising phase gave a slope of 48.6±5.7 s^-1^ (*n* = 5 responses in 5 cells to flashes delivering 1.85·10^5^ or 3.76·10^5^ photons/μm^2^).

Characteristic features of light adaptation in green cones were measured by delivering flashes of light in the absence or in presence of a background of light. A step of light caused a fast decline in current, followed by a much slower recovery to a steady-state level (Fig 5A). The step response amplitude slightly declined in the course of this recording (Fig 5A) because, in darkness, the time interval in between the steps was not long enough to let the cell fully recover its dark state. Despite the large amount of light absorbed by the cone, the recordings were quite stable, as exemplified by the recording of Fig 5. Indeed, the waveform of the average response to three consecutive flashes delivered in the dark and during the step of light at the beginning (responses under the *black* bars, Fig 5A; *black* traces, Fig 5B), in the middle (*green*) and at the end (*red*) of the recording were almost identical. The average flash responses in the presence of a background of light were smaller and faster in respect to the ones recorded without any adapting light (Fig 5C and 5D). For example, the background of 1.3·10^5^ photons/(μm^2^·s) suppressed 63±9% of the dark current (10 steps averaged, *n* = 3) and reduced the sensitivity as follows: a flash delivering 4.89·10^3^ photons/μm^2^ in the dark suppressed ~70±4% of the current (274 responses averaged, *n* = 25), while a flash twice as strong delivered on the background suppressed ~45±12% of the current (27 responses averaged, *n* = 3; Fig 5C). Due to the desensitization extent, it was possible to compare only the response to saturating flashes delivered in the dark and on a background of light (Fig 5D).

## Discussion

The whole-cell patch-clamp recordings from zebrafish green cones performed with pressure-polished pipettes had reproducible sensitivity, waveform photoresponse kinetics and light adaptation. We therefore conclude that the light triggered enzyme cascade was not perturbed by these pipettes which, at the same time, enabled fast loading of exogenous molecules by positioning perfusion tubes very close to the pipette tip.

A peculiar feature of the light responses to supersaturating flashes is the very fast kinetics of their rising phase produced by a burst of PDE activity able, within ~12 ms, to hydrolyze most of the intracellular cGMP pool (Fig 4D). This could arise from the small volume of the cone outer segment [34] however, the possibility that other, less well understood mechanisms could account for these fast kinetics cannot be ruled out. The frequency of encounters between the photoactivated visual pigment rhodopsin (or cone opsin) and the cognate G-protein transducin has been considered a major factor in determining the slope of the response rising phase, i.e. the cascade activation kinetics [34]. It is however possible that, in the dark, dimers of rhodopsin may interact with transducin by forming preassembled complexes [29, 35], increasing the probability of encounters between these proteins. This would obviously accelerate photoresponse kinetics, a notion consistent with the rapidity of the observed response rising phase, but the underlying hypothesis is still being debated [36–38].

Another interesting feature of the flash response waveform is the cell to cell difference of its recovery phase (single vs dual kinetic components, Fig 4B). Dual kinetic recovery was particularly evident for the responses to flashes from moderate (i.e. suppressing above 20% of dark current, Fig 4A and 4B) to just saturating intensities. On the other hand, dim flashes (Fig 6A) or supersaturating flashes (Fig 4A) always elicited response recoveries with a single kinetic component. This suggests that the dual component recovery occurs when the transient fall in intracellular Ca^2+^ elicited by the flash is significant, but at the same time the flash intensity is low enough to allow the cascade (i.e. the phosphodiesterase) to be quickly inactivated. Therefore, the difference between the two recovery phases may originate from different intracellular Ca^2+^ dynamics and this would be detected by several Ca^2+^ sensor proteins regulating the phototransduction cascade at various levels (see Introduction), collectively acting as a negative feedback. It is possible to mathematically demonstrate that the difference between single and dual kinetic component response may simply be due to different cell Ca^2+^ buffering capacity, as explained below.

Consider a system that produces a “photoresponse” *x*_*s*_ *(t)* described by the Poisson equation [39]:

$$x_{s}\left(t\right)=A_{s}t^{3}e^{\frac{-t}{\tau }}$$

where *A*_*s*_ and *τ* are constants (6*A*, *red* and *orange* traces; 6*C* and *D*, *red* traces). This equation is intended to fit the smallest average response to a flash (intensity: 1.14·10^2^ photons/μm^2^; Fig 6A, noisy traces) where the changes in intracellular Ca^2+^ are minimized, and thus the intervention of the various Ca^2+^-dependent feedback pathways are minimized as well. The fitting (Fig 6A, *orange* trace) is quite accurate for the fast responses (as the one in Fig 6A, *grey dotted* noisy trace, produced by the same cell that generated the *red* responses in Fig 4B). However, no values of *A*_*s*_ and *τ* are able to fit the entire slow response waveform: the falling phase of *x*_*o*_ *(t)* (Fig 6A, *red* trace) is always faster than the experimental responses (as the one in Fig 6A, *black dotted* noisy trace, from the same cell in Fig 4A and 4B, *black* traces). These slow responses can be fitted by a different expression for *x*_*s*_ *(t)* but, for the sake of simplicity, the fitting illustrated in Fig 6B (*red* trace) is good enough for the argument that follows. Let’s assume that the photoresponse to a strong stimulus is described by the same equation and same *τ* as the response to the dimmest stimulus, but with a larger *A*_*s*_. Let’s also assume that this “larger” photoresponse is the “input” of a system regulated through a negative feedback mechanism (Fig 5B) aimed at reducing photoreceptor sensitivity and thus extend its dynamic range. Let *H* be the gain and *G* the amount of “regulated photoresponse” (or “output”), *x*_*o*_ *(t)*, subtracted (i.e. negatively “feedbacked”) from the input. The more intense the light absorbed by the photoreceptor, the larger the decline in intracellular Ca^2+^ and, consequently, the larger parameter *G* will be. A Ca^2+^ buffer introduces a delay, *Δ*, between the time in which an intracellular Ca^2+^ change takes place and the time in which the Ca^2+^ sensor proteins detect it. Let *x*_*oΔ*_ *(t)* be the delayed output that is now subtracted from the input, as a consequence of the delay *Δ* introduced in the feedback path by the buffer [21]. It follows that:

$$x_{o}\left(t\right)=H*\left[x_{s}\left(t\right)-x_{o\Delta }\left(t\right)*G\right]$$

In the absence of any delay, the amplitude of the regulated responses, *x*_*o*_ *(t)* (Fig 6C and 6D, *brown* traces) is just scaled down when compared to the un-regulated response, *x*_*s*_ *(t)* (Fig 6C and 6D, *red* traces), if:

H(1−G)<1

To fit two representative experimental photoresponses (of Fig 4B) to the same flash, one slow and recovering with a dual component kinetics (Fig 6C, *black dotted* trace), and one fast and recovering with a single component kinetics (Fig 6D, *black dotted* trace), it is necessary to set *Δ*≠0. This accelerates the theoretical photoresponse recovery which exhibits a dual (Fig 6C, *green* trace) or single kinetics component (Fig 6D, *green* trace), depending upon the values of *τ* and *Δ*. The former is constrained by the fit to the two responses to the dimmest flash (Fig 6A, noisy traces, same two cells of Fig 4B) while the latter is adjusted to optimize the fit to the experimental traces. Notably, the fitting to the fast response is still good, while the fit to the slow response (with a dual component recovery) still has a falling phase that is faster than the experimental one. Eventually, as *Δ* is made larger, i.e. the cell’s buffering capacity is increased, responses exhibit damped oscillations (Fig 6C and 6D, *blue* traces), as observed in other cone species, where *Δ* was experimentally increased by incorporating into the cone millimolar amounts of the fast Ca^2+^ buffer BAPTA [40]. The oscillations are larger and more persistent if *G* and/or *Δ* are increased; the frequency of the oscillations become smaller as *Δ* is made larger. It is important to emphasize that the dramatic changes in the photoresponse recovery waveform, produced by *Δ*, is independent by the nature of the feedback (i.e. which element of the transduction system is affected by the decline in intracellular Ca^2+^). Notably, the rising phase of the delayed regulated response is faster than the one with no delay (leaving all other parameters unchanged). In general, for a receptor to have a large operative range, its sensitivity must be progressively reduced as stimulus intensity is increased, and this requires the presence of a negative feedback. However, negative feedback compresses the entire response while, instead, a delay provides the same initial fast rising phase as the unregulated response but avoids the later response saturation. The advantage of this strategy is obvious: the survival of an animal is ultimately determined by the speed with which its photoreceptors detect a predator in dim or in strong light. It is possible that, in general, the response kinetics of any signal transduction cascade, regulated through a negative feedback by one or more intracellular messengers, is tuned by the capacity of cytosol to buffer this/these messenger/s, always ensuring a fast response onset for a wide range of stimulus intensities.

Interestingly, the damped oscillations of the recovery phase are thought to be typical of primate cones. However, as pointed out by Cao and colleagues [41], this is not the case: indeed, some primate cones showed monotonic responses, while others, during the same recording, exhibited monotonic or biphasic responses to the same flash intensity. Cao and colleagues also showed that it was possible to switch from one kind of response to the other one by simply changing external Ca^2+^ concentration, strongly indicating that these two recovery types were generated by two different delays (*Δ*) in Ca^2+^ feedback. On the basis of the present findings, it may be that the intrinsic Ca^2+^-buffering capacity of the zebrafish cone was somewhat modified during these whole-cell recordings (for instance, by washing out a soluble Ca^2+^ buffer), transforming a dual component response recovery into a single component one or vice-versa. However, even in recordings lasting more than 20 min, the stability of the response waveform rules out any significant alteration in the concentration of any of the key components involved in shaping the photoresponse (besides the inevitable loss of a significant number of chromophore molecules as a consequence of very strong and repetitive light stimuli, producing the observed fall in receptor sensitivity; Fig 2). Moreover, in none of our recordings was a switch from single component to dual component response recovery and back ever observed, as in Cao and colleagues recordings. Rather, there was a large cell-to-cell variation in the kinetics of the photoresponse falling phase (Fig 4B) to the same flash intensity, but that did not change during the course of the recordings. Because there was no correlation between *R*_*a*_ and waveform kinetics, this cell-to-cell variation could not be due to a different *R*_*a*_ which would, in principle, give different Ca^2+^ dialysis. Indeed, in the course of five recordings, all lasting more than 7 min, *R*_*a*_ changed up to seven-fold (the smallest change was from 4.1 to 11.5 MΩ, the largest from 3.7 to 26 MΩ) but there was no change in the response waveform within this period of time. Finally, the recordings obtained with a pipette solution from which the Ca^2+^ contamination (~1 μM) had been removed using 25 μM of BAPTA (1,2-bis(o-aminophenoxy)ethane-N,N,N',N'-tetraacetic acid, K^+^ salt) were indistinguishable from the ones obtained in the absence of this Ca^2+^ chelator. This demonstrates that the strong cooperative Ca^2+^ regulation of guanylate cyclase, the influx of Ca^2+^ through the light-regulated channels, and the efflux of Ca^2+^ through the exchanger come together to form a powerful system to clamp the intracellular Ca^2+^ concentration (and the dark current) to its physiological value. Indeed, to contrast this system and finally affect the photoresponse recovery kinetics, the photoreceptor outer segment must be dialyzed with hundreds of micromoles of BAPTA or of Ca^2+^ [14, 21]. All these results suggest that there is a cell-to-cell variation in Ca^2+^ dynamics and that this is primarily regulated by the Ca^2+^ buffering capacity. It is then possible that the photoreceptor Ca^2+^ dynamics is regulated at one or more stages by a still unknown mechanism. In different low vertebrate photoreceptor cells, the latter could be locked at different levels of activation, thus giving the observed differences in the recovery phase. With evolution, this mechanism could, in turn, be regulated in the same cell by specific physiological requirements, giving a different response to the same stimulus, as observed by Cao and colleagues.

## References

[1] Venkatakrishnan AJ, Deupi X, Lebon G, Tate CG, Schertler GF, Babu MM. Molecular signatures of G-protein-coupled receptors. Nature. 2013;494(7436):185–94. 10.1038/nature11896 23407534

[2] Kobilka BK. G protein coupled receptor structure and activation. Biochimica et biophysica acta. 2007;1768(4):794–807. 17188232 10.1016/j.bbamem.2006.10.021 PMC1876727

[3] Tischer D, Weiner OD. Illuminating cell signalling with optogenetic tools. Nature reviews Molecular cell biology. 2014;15(8):551–8. 10.1038/nrm3837 25027655 PMC4145075

[4] Takeuchi H, Kurahashi T. Photolysis of caged cyclic AMP in the ciliary cytoplasm of the newt olfactory receptor cell. The Journal of physiology. 2002;541(Pt 3):825–33. 12068043 10.1113/jphysiol.2002.016600 PMC2290348

[5] Ellis-Davies GC. Caged compounds: photorelease technology for control of cellular chemistry and physiology. Nature methods. 2007;4(8):619–28. 17664946 10.1038/nmeth1072 PMC4207253

[6] Howes KA, Pennesi ME, Sokal I, Church-Kopish J, Schmidt B, Margolis D, et al GCAP1 rescues rod photoreceptor response in GCAP1/GCAP2 knockout mice. The EMBO journal. 2002;21(7):1545–54. 11927539 10.1093/emboj/21.7.1545 PMC125366

[7] Peachey NS, Ball SL. Electrophysiological analysis of visual function in mutant mice. Documenta ophthalmologica Advances in ophthalmology. 2003;107(1):13–36. 12906119 10.1023/a:1024448314608

[8] Chang B, Hawes NL, Hurd RE, Davisson MT, Nusinowitz S, Heckenlively JR. Retinal degeneration mutants in the mouse. Vision research. 2002;42(4):517–25. 11853768 10.1016/s0042-6989(01)00146-8

[9] Calvert PD, Krasnoperova NV, Lyubarsky AL, Isayama T, Nicolo M, Kosaras B, et al Phototransduction in transgenic mice after targeted deletion of the rod transducin alpha -subunit. Proceedings of the National Academy of Sciences of the United States of America. 2000;97(25):13913–8. 11095744 10.1073/pnas.250478897 PMC17675

[10] O'Sullivan GJ, O'Tuathaigh CM, Clifford JJ, O'Meara GF, Croke DT, Waddington JL. Potential and limitations of genetic manipulation in animals. Drug discovery today Technologies. 2006;3(2):173–80. 10.1016/j.ddtec.2006.06.005 24980405

[11] Chen CK. The vertebrate phototransduction cascade: amplification and termination mechanisms. Reviews of physiology, biochemistry and pharmacology. 2005;154:101–21. 16634148 10.1007/s10254-005-0004-0

[12] Lamb TD, Pugh EN Jr. Phototransduction, dark adaptation, and rhodopsin regeneration the proctor lecture. Investigative ophthalmology & visual science. 2006;47(12):5137–52. 17122096 10.1167/iovs.06-0849

[13] Arshavsky VY, Lamb TD, Pugh EN Jr. G proteins and phototransduction. Annual review of physiology. 2002;64:153–87. 11826267 10.1146/annurev.physiol.64.082701.102229

[14] Rispoli G. Calcium regulation of phototransduction in vertebrate rod outer segments. Journal of photochemistry and photobiology B, Biology. 1998;44(1):1–20. 9745724 10.1016/S1011-1344(98)00083-9

[15] Leskov IB, Klenchin VA, Handy JW, Whitlock GG, Govardovskii VI, Bownds MD, et al The gain of rod phototransduction: reconciliation of biochemical and electrophysiological measurements. Neuron. 2000;27(3):525–37. 11055435 10.1016/s0896-6273(00)00063-5

[16] Wen XH, Dizhoor AM, Makino CL. Membrane guanylyl cyclase complexes shape the photoresponses of retinal rods and cones. Frontiers in molecular neuroscience. 2014;7:45 10.3389/fnmol.2014.00045 24917784 PMC4040495

[17] Sakurai K, Chen J, Kefalov VJ. Role of guanylyl cyclase modulation in mouse cone phototransduction. The Journal of neuroscience: the official journal of the Society for Neuroscience. 2011;31(22):7991–8000. 21632921 10.1523/JNEUROSCI.6650-10.2011 PMC3124626

[18] Invergo BM, Dell'Orco D, Montanucci L, Koch KW, Bertranpetit J. A comprehensive model of the phototransduction cascade in mouse rod cells. Molecular bioSystems. 2014;10(6):1481–9. 10.1039/c3mb70584f 24675755

[19] Hamer RD, Nicholas SC, Tranchina D, Lamb TD, Jarvinen JL. Toward a unified model of vertebrate rod phototransduction. Visual neuroscience. 2005;22(4):417–36. 16212700 10.1017/S0952523805224045 PMC1482458

[20] Invergo BM, Montanucci L, Koch KW, Bertranpetit J, Dell'orco D. Exploring the rate-limiting steps in visual phototransduction recovery by bottom-up kinetic modeling. Cell communication and signaling: CCS. 2013;11(1):36. 23693153 10.1186/1478-811X-11-36 PMC3732082

[21] Moriondo A, Rispoli G. A step-by-step model of phototransduction cascade shows that Ca2+ regulation of guanylate cyclase accounts only for short-term changes of photoresponse. Photochemical & photobiological sciences: Official journal of the European Photochemistry Association and the European Society for Photobiology. 2003;2(12):1292–8. 10.1039/b303871h 14717223

[22] Gestri G, Link BA, Neuhauss SC. The visual system of zebrafish and its use to model human ocular diseases. Developmental neurobiology. 2012;72(3):302–27. 10.1002/dneu.20919 21595048 PMC3202066

[23] Kimmel CB, Ballard WW, Kimmel SR, Ullmann B, Schilling TF. Stages of embryonic development of the zebrafish. Developmental dynamics: an official publication of the American Association of Anatomists. 1995;203(3):253–310. 8589427 10.1002/aja.1002030302

[24] Kawamura S, Tachibanaki S. Explaining the functional differences of rods versus cones. Wiley Interdisciplinary Reviews: Membrane Transport and Signaling. 2012;1(5):675–83.

[25] Cilluffo MC, Matthews HR, Brockerhoff SE, Fain GL. Light-induced Ca2+ release in the visible cones of the zebrafish. Visual neuroscience. 2004;21(4):599–609. 15579223 10.1017/S0952523804214092

[26] Leung YT, Fain GL, Matthews HR. Simultaneous measurement of current and calcium in the ultraviolet-sensitive cones of zebrafish. The Journal of physiology. 2007;579(Pt 1):15–27. 17124271 10.1113/jphysiol.2006.120162 PMC2075373

[27] Takemoto N, Tachibanaki S, Kawamura S. High cGMP synthetic activity in carp cones. Proceedings of the National Academy of Sciences of the United States of America. 2009;106(28):11788–93. 10.1073/pnas.0812781106 19556550 PMC2710672

[28] Benedusi M, Aquila M, Milani A, Rispoli G. A pressure-polishing set-up to fabricate patch pipettes that seal on virtually any membrane, yielding low access resistance and efficient intracellular perfusion. European biophysics journal: EBJ. 2011;40(11):1215–23. 10.1007/s00249-011-0727-y 21761372

[29] Dell'Orco D, Koch KW. A dynamic scaffolding mechanism for rhodopsin and transducin interaction in vertebrate vision. The Biochemical journal. 2011;440(2):263–71. 10.1042/BJ20110871 21843151

[30] Avdesh A, Chen M, Martin-Iverson MT, Mondal A, Ong D, Rainey-Smith S, et al Regular care and maintenance of a zebrafish (Danio rerio) laboratory: an introduction. Journal of visualized experiments: JoVE. 2012 (69):e4196 10.3791/4196 23183629 PMC3916945

[31] Aquila M, Benedusi M, Fasoli A, Rispoli G. Pressure-polished borosilicate pipettes are "universal sealer" yielding low access resistance and efficient intracellular perfusion. Methods Mol Biol. 2014;1183:279–89. 10.1007/978-1-4939-1096-0_18 25023316

[32] Baylor DA, Lamb TD, Yau KW. Responses of retinal rods to single photons. The Journal of physiology. 1979;288:613–34. 112243 PMC1281447

[33] Robinson J, Schmitt EA, Hárosi FI, Reece RJ, Dowling JE. Zebrafish ultraviolet visual pigment: absorption spectrum, sequence, and localization. Proc Natl Acad Sci U S A. 1993; 90:6009–12. 8327475 10.1073/pnas.90.13.6009 PMC46856

[34] Pugh EN Jr, Lamb TD. Chapter 5 Phototransduction in vertebrate rods and cones: Molecular mechanisms of amplification, recovery and light adaptation In: D.G. Stavenga WJD, Pugh EN, editors. Handbook of Biological Physics: North-Holland; 2000;183–255.

[35] Dell'Orco D, Schmidt H. Mesoscopic Monte Carlo simulations of stochastic encounters between photoactivated rhodopsin and transducin in disc membranes. The journal of physical chemistry B. 2008;112(14):4419–26. 10.1021/jp709963f 18345658

[36] Biedermann J, Ullrich A, Schoneberg J, Noe F. ReaDDyMM: Fast interacting particle reaction-diffusion simulations using graphical processing units. Biophysical journal. 2015;108(3):457–61. 10.1016/j.bpj.2014.12.025 25650912 PMC4317564

[37] Dell'Orco D, Koch KW. Transient complexes between dark rhodopsin and transducin: circumstantial evidence or physiological necessity? Biophysical journal. 2015;108(3):775–7. 10.1016/j.bpj.2014.12.031 25650944 PMC4317538

[38] Schoneberg J, Hofmann KP, Heck M, Noe F. Response to comment "Transient complexes between dark rhodopsin and transducin: circumstantial evidence or physiological necessity?" by D. Dell'Orco and K.-W. Koch. Biophysical journal. 2015;108(3):778–9. 10.1016/j.bpj.2014.12.030 25650945 PMC4317562

[39] Lamb TD, Baylor DA, Yau KW. The membrane current of single rod outer segments. Vision research. 1979;19(4):385 112774 10.1016/0042-6989(79)90099-3

[40] Matthews HR, Fain GL, Murphy RL, Lamb TD. Light adaptation in cone photoreceptors of the salamander: a role for cytoplasmic calcium. J Physiol. 1990;420:447–69. 2109062 10.1113/jphysiol.1990.sp017922 PMC1190059

[41] Cao LH, Luo DG, Yau KW. Light responses of primate and other mammalian cones. Proceedings of the National Academy of Sciences of the United States of America. 2014;111(7):2752–7. 10.1073/pnas.1400268111 24550304 PMC3932881

